# Post-traumatic Stress Disorder Symptoms and Quality of Life of COVID-19 Survivors at 6-Month Follow-Up: A Cross-Sectional Observational Study

**DOI:** 10.3389/fpsyt.2021.782478

**Published:** 2022-01-10

**Authors:** Liqun Huang, Xiaohua Xu, Lingjie Zhang, Danwen Zheng, Yuntao Liu, Bing Feng, Jiajun Hu, Qiaoli Lin, Xiaotu Xi, Qian Wang, Meixuan Lin, Xin Zhou, Zehui He, Heng Weng, Qiuying Deng, Banghan Ding, Jianwen Guo, Zhongde Zhang

**Affiliations:** ^1^The Second Clinical College, Guangzhou University of Chinese Medicine, Guangzhou, China; ^2^Department of Medical Administration, Hubei Provincial Hospital of Traditional Chinese and Western Medicine, Wuhan, China; ^3^Department of Emergency, The Second Affiliated Hospital of Guangzhou University of Chinese Medicine, Guangzhou, China; ^4^Department of Pharmacology of Traditional Chinese Medicine, The Second Affiliated Hospital of Guangzhou University of Chinese Medicine, Guangzhou, China; ^5^Department of Geriatrics, The Second Affiliated Hospital of Guangzhou University of Chinese Medicine, Guangzhou, China; ^6^Department of Clinical Epidemiology, The Second Affiliated Hospital of Guangzhou University of Chinese Medicine, Guangzhou, China; ^7^Department of Medical Administration, The Second Affiliated Hospital of Guangzhou University of Chinese Medicine, Guangzhou, China; ^8^Guangdong Provincial Key Laboratory of Research on Emergency in Traditional Chinese Medicine, Guangzhou, China

**Keywords:** COVID-19, survivors, clinical sequelae, post-traumatic stress disorder, quality of life (QoL)

## Abstract

**Background:** Post-traumatic stress disorder (PTSD) is the most common psychiatric sequelae among novel coronavirus disease (COVID-19) patients. The aim of this study was to determine the prevalence of PTSD symptoms, PTSD-related factors, and its relationship with quality of life at long-term follow-up in hospitalized COVID-19 survivors.

**Methods:** A cross-sectional study was undertaken to evaluate the health consequences of hospitalized COVID-19 survivors. All participants were interviewed face-to-face through a series of questionnaires: a researcher-developed symptom questionnaire, the Post-traumatic Stress Disorder Checklist–Civilian Version, the Generalized Anxiety Disorder 7-item, and the 36-item Short Form.

**Results:** A total of 574 participants were enrolled with an average age of 57 years. The median follow-up time post-discharge was 193.9 days (SD = 15.32). Among the participants, 77.9% of survivors presented with at least one symptom, where fatigue or muscle weakness (47.9%) was reported the most frequently, followed by chest distress (29.4%) and sleep difficulty (29.4%). The prevalence of PTSD was 11.15% [95% confidence interval (CI): 8.56, 13.73] with a cut-off score of 44. Factors such as respiratory symptoms [odds ratio (OR): 3.53; 95% CI: 1.68–7.42], anxiety (OR: 14.64; 95% CI: 7.09–30.21), and sleep difficulty (OR: 2.17; 95% CI: 1.14–4.16) were positively related to PTSD. Those COVID-19 survivors with potential PTSD had significantly lower quality of life than those without (*P* < 0.05).

**Conclusion:** Our study illustrated that a significant number of COVID-19 survivors were suffering from physical or mental distress to varying degrees at 6 months post-discharge. People with PTSD were more likely to experience persistent respiratory symptoms and sleep difficulty, as well as anxiety and a decreased quality of life. Such survivors require greater attention to their mental health, particularly the PTSD symptoms at the early phase, which may play an important role in the recovery of both the physical and psychological health of COVID-19 survivors.

## Introduction

The novel coronavirus disease (COVID-19) has spread rapidly throughout the world with millions of people infected by the severe acute respiratory syndrome coronavirus 2 (SARS-COV-2), while the clinical spectrum of SARS-COV-2 infection ranges from asymptomatic infection to life-threatening and fatal disease. An increasing body of literature suggests that a significant proportion of survivors will experience long-term sequelae from COVID-19, including cardiopulmonary consequences, neurocognitive impairment, and pulmonary dysfunction, as well as psychological sequelae (1–3).

Although a considerable number of studies have addressed the general medical sequelae and complications of COVID-19, a paucity of research has focused its lens on the effect of psychological sequelae in the post-illness stage of COVID-19. A systematic review and meta-analysis on severe acute respiratory syndrome coronavirus (SARS) and Middle East respiratory syndrome (MERS) survivors found an increased prevalence of psychological sequelae during both the illness and post-illness stages (4), including depression, anxiety, and post-traumatic stress disorder (PTSD). Previous epidemiological studies highlighted that infectious disease outbreaks such as SARS, MERS, and COVID-19 may increase the risk of future PTSD symptoms (5). The prevalence of PTSD symptoms is in the range of 3% among the general population, which increases to over 40% among the survivors of coronavirus (6, 7). Follow-up studies on Ebola virus disease and SARS have shown that PTSD symptoms were the most common psychological sequelae (8, 9). Moreover, despite most psychological sequelae fading out post-infection, PTSD may last for a prolonged period and become chronic (10, 11).

PTSD, a severe mental disorder, can be caused by experiencing a life-threatening or terrifying event (12) and is a risk factor of alcohol and substance abuse, poor mental health, and physical health consequences such as chronic pain, hypertension, obesity, and cardiovascular disease (13, 14). PTSD is also related to suicidal ideation and suicide attempts, whereby individuals experiencing PTSD symptoms are at a significantly higher risk of suicidal behavior (15, 16). In addition, PTSD symptoms may have a negative impact on both mental and physical functions, which can result in severe distress, disability, and reduced quality of life (QoL) (17). QoL is a multidimensional health outcome influenced by economic and social factors, life satisfaction, and the severity and stage of a disease (18) and has been regarded as the most significant predictor of stress symptoms (19). However, no studies to date have explored the relationship between PTSD symptoms and the long-term outcomes of QoL among COVID-19 survivors.

Some factors have been revealed to be associated with PTSD symptoms in short-term follow-up studies (20) on COVID-19 survivors, including illness severity (21, 22), female gender (23), depression and anxiety (24), pre-existing mental disorders (25), time elapsed post-discharge (26), and the burden of influenza-like symptoms (27). However, a number of common limitations such as small sample size, single study site, and short-term follow-up, as well as the lack of face-to-face interview, have limited the application of the data from these studies. Little is known about the long-term consequences of PTSD symptoms and the impact on QoL among COVID-19 survivors. Considering that COVID-19 hospitalized patients showed wide-ranging prevalence rates of PTSD symptoms and poor QoL at short-term follow-up, further and longer follow-up research is necessary to explore the potential risk factors for PTSD symptoms (28).

In order to reduce the negative consequences of PTSD symptoms, improve the recovery of COVID-19 survivors, and assist health authorities in developing effective strategies for the prevention of PTSD symptoms, it is of great significance to explore the pattern of PTSD symptoms, as well as the risk factors (29). Hence, this study aims to determine the prevalence of PTSD symptoms, as well as the PTSD-related factors of COVID-19 survivors at 6-month follow-up. Furthermore, the relationship between PTSD and QoL among COVID-19 survivors will also be explored.

## Methods

### Study Design and Participants

A cross-sectional follow-up study was conducted between August 28 and September 30, 2020, at Xin Hua Hospital (Hubei Provincial Hospital of Traditional Chinese and Western Medicine), one of the designated hospitals for patients with COVID-19 in Wuhan, Hubei province, China. This study forms part of a larger project on the health outcomes of patients affected by COVID-19 who required hospitalization at the beginning of the pandemic. We recruited COVID-19 survivors aged 18 years or older, who had been hospitalized in the designated isolation hospitals in Wuhan and who were discharged from January 17 to June 24, 2020. The following exclusion criteria were applied: (1) those unable to be contacted; (2) those who were unable to move freely due to concomitant osteoarthropathy or were immobile pre- or post-discharge due to diseases such as stroke or pulmonary embolism; (3) those living outside of Wuhan city; (4) those with a history of mental disorder prior to SARS-COV-2 infection; and (5) those who declined to participate. All subjects recruited met the uniform diagnostic criteria and discharge criteria according to the Diagnosis and Treatment Protocol for Novel Coronavirus Pneumonia (Trial Version 8) in China (General Office of the National Health Commission, 2020) (30). The Ethical Committee of Guangdong Provincial Hospital of Chinese Medicine reviewed and approved the study protocol (BF2020-205-01). Informed consent was obtained prior to any participation in the study.

### Information Sources

Data information was collected in person by trained medical doctors in the outpatient clinic of Xin Hua Hospital, including basic socio-demographic data (e.g., age, sex, location of residency, and marital status) and clinical characteristics (e.g., self-reported comorbidities and history of COVID-19). Then, a series of questionnaires were utilized to determine (1) the symptoms at follow-up (researcher-developed symptom questionnaire), (2) the symptoms of PTSD [Post-traumatic Stress Disorder Checklist–Civilian Version (PCL-C)], (3) the symptoms of anxiety [Generalized Anxiety Disorder 7-item (GAD-7)], and (4) the health-related QoL [36-item Short Form (SF-36)].

The disease severity of COVID-19 was characterized by the highest seven-category scale during the period of infection (termed the severity scale) (31), which consisted of the following categories: (1) not admitted to hospital, with a resumption of normal activities; (2) not admitted to hospital, but unable to resume normal activities; (3) admitted to hospital, but not requiring supplemental oxygen; (4) admitted to hospital and requiring supplemental oxygen; (5) admitted to hospital and requiring high-flow nasal cannula, non-invasive mechanical ventilation, or both; (6) admitted to hospital and requiring extracorporeal membrane oxygenation, invasive mechanical ventilation, or both; and (7) death. All subjects recruited in our study ranged from scale 3 to scale 6. For the sake of analysis, we classified the scale 3–5 as mild and 6 as severe.

The symptoms at follow-up were classified into five categories based on previous COVID-19-related reports, covering (1) general symptoms, such as fatigue or muscle weakness, sweating, myalgia or joint pain, and chills; (2) respiratory symptoms, including shortness of breath, chest distress, chest pain, cough, and sputum; (3) digestive symptoms, such as decreased appetite, abdominal distention or bloating, vomiting or nausea, and diarrhea; (4) neurological symptoms, including forgetfulness, hypoplasia, and hearing loss; and (5) psychosocial symptoms, such as PTSD, anxiety, sleep difficulty, and poor mental component summary (MCS) scores in the SF-36. All symptom data were acquired *via* the aforementioned questionnaires.

### Instruments

The PCL-C is a 17-item measure utilized to screen for PTSD. The instrument quantifies the resultant symptoms (32) and reflects the Diagnostic and Statistical Manual of Mental Disorders' (4th ed.) PTSD symptom criteria. Symptom severity rated on the Likert scale ranges from 1 (mild) to 5 (severe), and a total score is calculated for each item (33). The Chinese version of the PCL-C has shown high internal consistency and convergent validity (34). Since a score of 44 is the cut-off and is used as a screener for potential symptoms in the natural disaster setting in China, in this study, we refer to patients with a score ≥44 as having positive symptoms of PTSD (35).

The anxiety symptoms were assessed using GAD-7, a seven-item self-reporting instrument compiled by Spitzer et al. for screening generalized anxiety and symptom severity (36). Previous studies have shown GAD-7 to be a useful tool for evaluating the anxiety symptoms of the general population and HIV patients (37, 38). The validity and reliability of the Chinese version of GAD-7 were tested in clinical settings, and Cronbach's α was 0.91 (39). The seven items of the GAD-7 are summed to produce a total score ranging from 0 to 21, with higher values indicating a greater degree of anxiety. A score of 10 is considered an appropriate cut-off to identify generalized anxiety disorder (40).

QoL was estimated using the Chinese version of the 36-item Short Form health questionnaire (41). The SF-36 is a reliable instrument to evaluate the patient QoL by measuring eight dimensions of health status: physical function, physical role, bodily pain, general health, vitality, social functioning, emotional role, and mental health. According to the standardized scoring of measures of physical and mental factors, the total scores of the SF-36, including the physical component summary (PCS) and the MCS, have been converted into *T* scores, with a mean of 50 and a standard deviation of 10 for the normal population (42). The scoring range is 0–100 for each domain, with higher scores representing enhanced QoL, while a total score below 50 indicates poor physical or mental health (43).

### Statistical Analysis

All data were analyzed with SPSS (v.18.0). The normality of continuous variables was examined by the *P*–*P* plot. The demographic and clinical variables between PTSD and non-PTSD were compared using univariate analyses, chi-square test and Fisher's exact test were employed to compare the categorical variables, and two independent samples' *T*-test and Mann–Whitney *U* test were used to compare normally and non-normally distributed continuous variables, respectively. Binary logistic regression analysis with the “enter” method was utilized to explore the independent correlates of PTSD. PTSD was the dependent variable, while variables with a *P-*value of <0.1 in univariate analyses and those having a potential correlation with PTSD symptoms based on clinical knowledge were entered as independent variables. The significance level was set as *P* < 0.05 (two-tailed).

## Results

### Socio-Demographic and Clinical Characteristics

**Figure 1:**
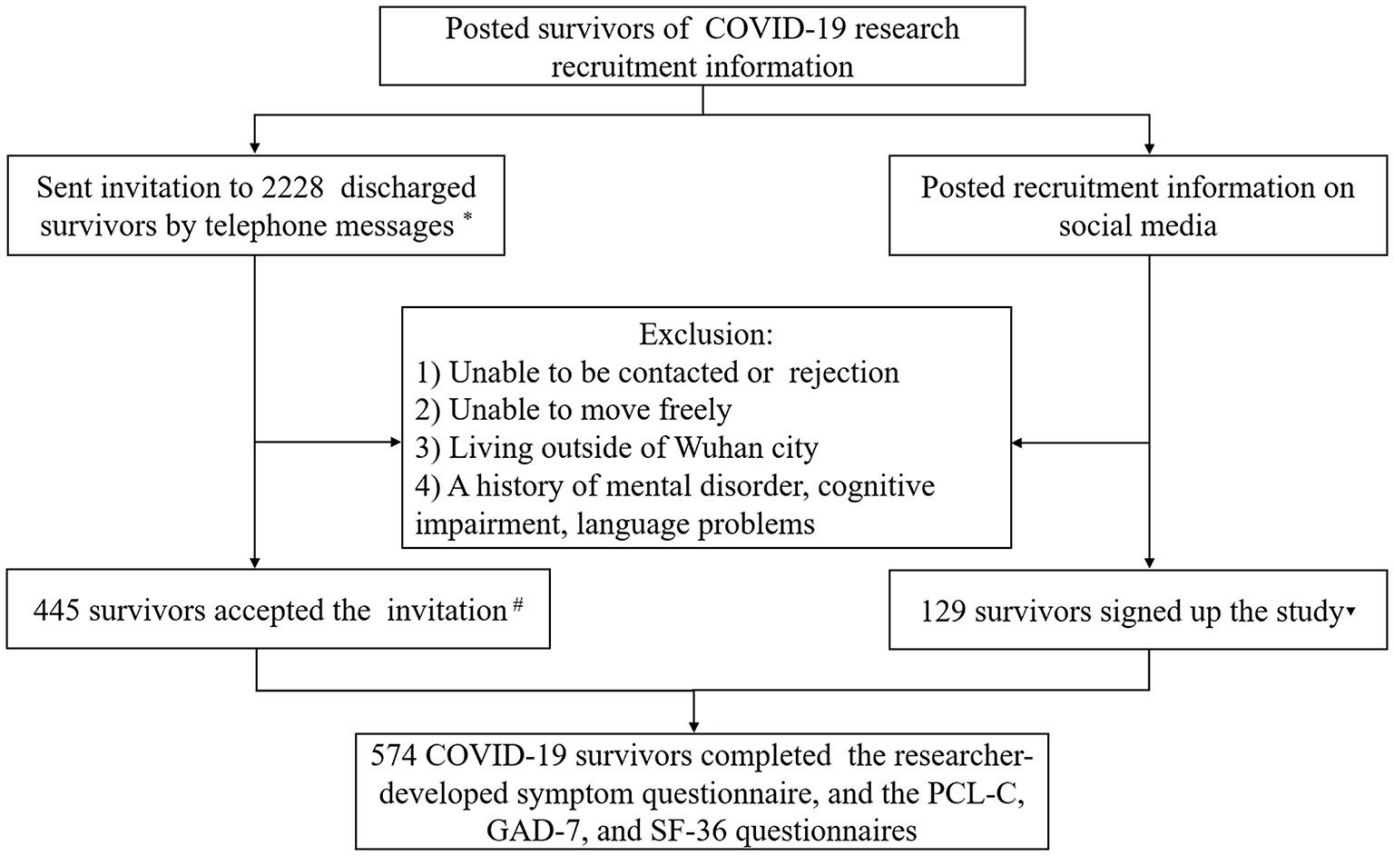
Flow chart of COVID-19 survivor recruitment. *The breakdown of survivors is as follows: from Hubei Provincial Hospital of Traditional Chinese and Western Medicine (*N* = 682), Wuhan's Leishenshan Hospital (*N* = 1,014), and Wuhan's Hankou Hospital (*N* = 532). ^#^The breakdown of survivors is as follows: from Hubei Provincial Hospital of Traditional Chinese and Western Medicine (*N* = 197), Wuhan's Leishenshan Hospital (*N* = 165), and Wuhan's Hankou Hospital (*N* = 83). ^▾^The breakdown of survivors is as follows: from Wuhan's Huoshenshan Hospital (*N* = 70), Wuhan Jinyintan Hospital (*N* = 6), Taikang Tongji (Wuhan) Hospital (*N* = 12), Wuhan Asia Heart Hospital (*N* = 5), Wuhan No. 1 Hospital (*N* = 4), Wuhan No. 3 Hospital (*N* = 3), Wuhan No. 4 Hospital (*N* = 3), Wuhan No. 9 Hospital (*N* = 3), Renmin Hospital of Wuhan University (*N* = 3), Wuhan Wuchang Hospital (*N* = 2), Central Hospital of Wuhan (*N* = 2), Wuhan Hanyang Mobile Cabin Hospital (*N* = 7), Wuhan Jianghan Mobile Cabin Hospital (*N* = 1), Wuhan Dongxihu District Mobile Cabin Hospital (*N* = 2), Wuhan Union Hospital of China (*N* = 4), and Union Jiangbei Hospital (*N* = 2).

In total, 2,228 COVID-19 survivors who received treatment in Xinhua Hospital, Hankou Hospital, Leishenshan Hospital, and Huoshenshan Hospital in Wuhan, China, during the COVID-19 acute phase received our telephone invitation, of which 445 patients met the inclusion criteria and participated in the assessment. Furthermore, 129 COVID-19 survivors treated in other hospitals during the acute phase in Wuhan were also recruited through our social media channels and completed the assessment. Therefore, 574 COVID-19 survivors completed the study (Figure 1). The socio-demographic and clinical characteristics are presented in Table 1. The mean age of the enrolled participants is 57.67 [SD = 11.42 years; 226 (39.4%) males and 348 females (60.6%)]. The most common comorbidities are hypertension (27.2%), followed by diabetes (14.6%) and cardiovascular disease (6.4%). The mean duration of hospital stay, from symptom onset to admission, and from discharge to follow-up visit, is 18.49 (SD = 12.17), 29.75 (SD = 16.30), and 193.99 days (SD = 15.32), respectively.

**Table 1:** Demographic and clinical characteristics of the whole sample and PTSD subgroups.

**Variable**	**Total**	**PTSD symptoms**		***P*-value**
	**(*N* = 574)**	**Yes (*N* = 64)**	**No (*N* = 510)**	
	***N* (%)**	***N* (%)**	***N* (%)**	
**Sex**				
Male	226 (39.4)	16 (25.0)	210 (41.2)	**0.013**
Female	348 (60.6)	48 (75.0)	300 (58.8)	
Severe COVID-19 infection	113 (19.7)	16 (25.0)	97 (19.0)	0.257
**Comorbidity**				
Total	245 (42.7)	26 (40.6)	219 (42.9)	0.637
Hypertension	156 (27.2)	16 (25.0)	140 (27.5)	0.678
Diabetes	84 (14.6)	6 (9.4)	78 (15.3)	0.207
Coronary heart disease	37 (6.4)	4 (6.3)	33 (6.5)	1.000^a^
Chronic lung disease	22 (3.8)	4 (6.3)	18 (3.5)	0.130^a^
Chronic kidney disease	7 (1.2)	0	7 (1.4)	1.000^a^
Chronic liver disease	12 (2.1)	2 (3.1)	10 (2.0)	0.633^a^
Cerebrovascular disease	13 (2.3)	3 (4.7)	10 (2.0)	0.168^a^
Cancer	16 (2.8)	0	16 (2.8)	0.238^a^
Clinical sequelae at discharge	381 (66.4)	54 (84.4)	327 (64.1)	**0.001**
Clinical sequelae at follow-up	447 (77.9)	64 (100.0)	383 (75.1)	**<0.001**
	**Mean (SD)**	**Mean (SD)**	**Mean (SD)**	***P*** **-value**
Age (years)	57.67 (11.42)	57.25 (10.62)	57.72 (11.53)	0.757^b^
Time from symptom onset to admission (days)	29.75 (16.30)	31.90 (18.09)	29.48 (16.06)	0.282^b^
Length of hospital stay (days)	18.49 (12.17)	19.91 (12.39)	18.31 (12.15)	0.324^b^
Time from discharge to follow-up (days)	193.99 (15.32)	192.31 (13.95)	194.21 (15.48)	0.352^b^

a *Fisher's exact test*.

b *Two independent-samples T-test*.

*Bold values: P < 0.05*.

At follow-up, 77.9% of the survivors reported at least one symptom (i.e., any one symptom mentioned in the questionnaires, either physical or psychological), and a higher percentage was observed in the PTSD group compared to the non-PTSD group (100% vs. 75.1%). General symptoms (e.g., fatigue or muscle weakness, sweating, myalgia or joint pain, and chills) and respiratory symptoms (e.g., cough, sputum, and shortness of breath) are the most common manifestations (Table 2).

**Table 2:** Clinical sequelae of COVID-19 survivors.

**Variable**	**Total**	**PTSD symptoms**	**Non-PTSD**	***P*-value**
	**(*N* = 570)**	**(*N* = 64)**	**(*N* = 510)**	
	***N* (%)**	***N* (%)**	***N* (%)**	
**General symptoms**				
Any symptom	321 (55.9)	53 (82.8)	268 (52.5)	**<0.001**
Fatigue or muscle weakness	275 (47.9)	49 (76.6)	226 (44.3)	**<0.001**
Sweating	78 (13.6)	19 (29.7)	59 (11.6)	**<0.001**
Myalgia or joint pain	53 (9.3)	9 (14.1)	44 (8.6)	0.157
Chills	114 (19.9)	21 (32.8)	93 (18.2)	**0.012**
**Respiratory symptoms**				
Any symptom	265 (46.2)	50 (78.1)	215 (42.2)	**<0.001**
Shortness of breath	165 (28.7)	38 (59.4)	127 (24.9)	**<0.001**
Chest distress	169 (29.4)	36 (56.3)	133 (26.1)	**<0.001**
Chest pain	49 (8.5)	10 (15.6)	39 (7.6)	**0.015**
Cough	66 (11.5)	57 (11.2)	9 (14.1)	0.495
Expectoration (sputum)	35 (6.1)	8 (12.5)	27 (5.3)	**0.045^a^**
**Digestive symptoms**				
Any symptom	84 (14.6)	21 (32.8)	63 (12.4)	**<0.001**
Decreased appetite	44 (7.7)	16 (25.0)	28 (5.5)	**<0.001^a^**
Abdominal distention or bloating	33 (5.7)	7 (10.9)	26 (5.1)	**0.034^a^**
Vomiting or nausea	14 (2.4)	3 (4.7)	11 (2.2)	0.198^a^
Diarrhea	26 (4.5)	7 (10.9)	19 (3.7)	**0.018^a^**
**Neurologic symptoms**				
Any symptom	75 (13.1)	10 (15.6)	65 (12.7)	0.519
Forgetfulness	69 (12.0)	8 (12.5)	61 (12.0)	0.900
Hypopsia	8 (1.4)	2 (3.1)	6 (1.2)	0.210^a^
Hearing loss	9 (1.6)	3 (4.7)	6 (1.2)	0.680^a^
**Psychosocial symptoms**				
Any symptom	201 (35.0)	53 (82.8)	148 (29.0)	**<0.001**
Sleep difficulty	169 (29.4)	36 (56.3)	133 (26.1)	**<0.001**
GAD-7, score ≥10	62 (10.8)	33 (51.6)	29 (5.7)	**<0.001**
**SF-36**				
MCS, score <50	438 (76.3)	62 (96.9)	376 (73.7)	**<0.001**
PCS, score <50	216 (37.6)	44 (68.8)	172 (33.7)	**<0.001**
	**Mean (SD)**	**Mean (SD)**	**Mean (SD)**	***P*** **-value**
PCL-C score	28.45 (11.07)	52.03 (7.90)	25.49 (7.18)	**<0.001^b^**
GAD-7 score	3.86 (4.65)	11.13 (5.52)	2.95 (3.63)	**0.001^b^**
**SF-36**				
PCS score	51.66 (7.80)	46.40 (8.24)	52.32 (7.49)	**0.001^b^**
MCS score	39.76 (11.35)	27.11 (8.29)	41.35 (10.68)	**0.001^b^**

a *Fisher's exact test*.

b *Two independent-samples T-test*.

*Bold values: P < 0.05*.

*SD, standard deviation*.

According to the PCL-C score, the survivors were divided into a PTSD group (PCL-C ≥ 44) and non-PTSD group (PCL-C < 44). The prevalence of PTSD symptoms is 11.15% [95% confidence interval (CI): 8.56, 13.73], with a mean total score of 52.03 (SD = 7.90) in the PTSD group and 25.49 (SD = 7.18) in the non-PTSD group. The prevalence of anxiety (GAD-7 score ≥ 10) is 10.8% (95% CI: 8.25, 13.33), while the mean total scores of the GAD-7, PCS, and MCS are 3.86 (SD = 4.65), 51.66 (SD = 7.80), and 39.76 (SD = 11.35), respectively.

According to the results of the univariate analyses (Tables 1, 2), we found that female survivors (*P* = 0.013); those who were with ongoing symptoms at discharge (*P* = 0.001); those who struggled with long-lasting symptoms at follow-up (*P* < 0.001), including general (*P* < 0.001), respiratory (*P* < 0.001), and digestive (*P* < 0.001) symptoms; those who experienced difficulty in sleeping (*P* < 0.001); and those who had a lower score in GAD-7 (*P* < 0.001), PCS (*P* **<** 0.001), or MCS (*P* < 0.001) were more likely to be accompanied by PTSD symptoms.

Table 3 shows the mean score of the eight dimensions of the SF-36 and their relation with PTSD, where all dimensions obtained statistically lower scores in the PTSD group than the non-PTSD counterpart. The most affected dimension was emotional role (mean difference: −43.15; 95% CI: −54.82, −31.47).

**Table 3:** Quality-of-life scores for PTSD and dimensions (mean ± SD).

**Variable**	**PTSD (*N* = 64)**	**Non-PTSD (*N* = 510)**	**MD**	**95% CI**
	**Mean (SD)**	**Mean (SD)**		
Physical function	76.87 (14.04)	88.80 (12.13)	−11.93	**(−15.15**, **−8.71)**
Physical role	17.96 (37.39)	58.38 (48.80)	−40.41	**(−52.83**, **−27.99)**
Body pain	76.93 (28.68)	88.66 (21.26)	−11.72	**(−17.51**, **−5.94)**
General health	41.68 (19.31)	63.18 (20.58)	−21.49	**(−26.82**, **−16.17)**
Vitality	42.81 (18.97)	67.47 (20.39)	−24.65	**(−29.92**, **−19.38)**
Social function	32.03 (34.27)	63.06 (35.50)	−31.03	**(−40.25**, **−21.82)**
Emotional role	19.27 (35.53)	62.42 (45.84)	−43.15	**(−54.82**, **−31.47)**
Mental health	38.75 (14.46)	58.63 (16.25)	−19.87	**(−24.06**, **−15.70)**

*Bold values refer to P < 0.05*.

*CI, confidence interval; SD, standard deviation; MD, mean difference*.

Table 4 presents the results of the binary logistic regression analyses, where PTSD symptoms were positively associated at follow-up with respiratory symptoms [odds ratio (OR) = 3.53; 95% CI = 1.68–7.42, *P* < 0.01) and anxiety (OR = 14.64; 95% CI = 7.09–30.21, *P* < 0.001), as well as sleep difficulty (OR = 2.17, 95% CI = 1.14–4.16, *P* = 0.019).

**Table 4:** Factors associated with PTSD: Binary analyses.

**Variable**	***b* coefficient (SE)**	***P*-value**	**Odds ratio (95% CI)**
Sex, male	0.48 (0.37)	0.196	1.61 (0.78, 3.31)
Age (≥65)	0.13 (0.37)	0.731	1.14 (0.55, 2.37)
Severe COVID-19 infection	0.53 (0.41)	0.191	1.71 (0.77, 3.80)
Time from discharge to follow-up (≤ 180 days)	−0.69 (0.39)	0.780	0.50 (0.23, 1.08)
Comorbidity	−0.27 (0.35)	0.443	0.77 (0.39, 1.51)
Clinical sequelae at discharge	0.03 (0.44)	0.946	1.03 (0.44, 2.43)
**Clinical sequelae at follow-up**			
General symptoms	0.71 (0.42)	0.094	2.02 (0.89, 4.61)
Respiratory symptoms	1.26 (0.38)	**0.001**	**3.53 (1.68, 7.42)**
Digestive symptoms	0.55 (0.38)	0.146	1.73 (0.83, 3.62)
Neurologic symptoms	−0.14 (0.47)	0.77	0.87 (0.34, 2.19)
Anxiety	2.68 (0.37)	**<0.001**	**14.64 (7.09, 30.21)**
Sleep difficulty	0.78 (0.33)	**0.019**	**2.17 (1.14, 4.16)**

*Bold values refer to P < 0.05*.

*CI, confidence interval*.

## Discussion

The COVID-19 pandemic, which has infected over 250 million people and resulted in over five million deaths, has presented a formidable global challenge in terms of public health. With the rising number of COVID-19 patients being discharged, studies are increasingly focusing on the subsequent health outcomes of COVID-19 survivors after discharge, both physical and psychological, so did our research.

In our study, we found that 77.9% of COVID-19 survivors were suffering from at least one ongoing symptom 6 months after COVID-19 discharge, which is similar to that reported by Huang et al. (3), who, in their follow-up of 1,733 previously hospitalized COVID-19 patients, found that 76% of survivors had experienced at least one residual symptom 6 months after diagnosis. Moreover, we found that of all the residual symptoms, fatigue or muscle weakness was reported the most frequently,followed by chest distress and sleep difficulty. These results are closely consistent with other reports. For example, Huang et al. (3) also reported that fatigue or muscle weakness and sleep difficulties were the most common symptoms at the 6-month follow-up. In another study, Taylor et al. found that at an average 18 weeks post-acute infection, the COVID-19 survivors had a median of two ongoing physical complaints, with fatigue the most common (44). All these data illustrate that the majority of COVID-19 survivors suffer from at least one ongoing symptom at 3–6 months follow-up, with fatigue and sleep difficulty the most frequently reported symptoms. During previous severe coronavirus outbreaks, 40.3% of survivors suffered from chronic fatigue at a mean follow-up of 41.3 months (45). While one reason for the high rate of comorbidity at follow-up may be due to the pathogenic specificity of coronavirus (46), the clinical treatment in the acute phase also plays an important role. In a 12-year follow-up study, Wu et al. (47) found that SARS survivors had a higher risk of lung susceptibility to infections, tumors, cardiovascular disorders, and abnormal glucose metabolism compared to the healthy controls. These negative results are significantly related to the high-dose pulses of methylprednisolone in acute infection.

Beyond physical symptoms, the effects of SARS-COV-2 infection upon mental health are equally important (48). In a 1-month follow-up of COVID-19 survivors, the incidence of PTSD and anxiety was as high as 28% and 42%, respectively (49). A research by D'Cruz et al. (50) showed a rate of positive screening for anxiety, depression, and PTSD at 22, 18, and 25%, respectively, at a mean follow-up of 61 days. Moreover, Tarsitani et al. (25) reported a PTSD prevalence of 10.4% among pre-hospitalized (i.e., those who were hospitalized in an acute phase of COVID-19, but then recovered and were discharged) COVID-19 survivors at 3-month follow-up. In another 4-month follow-up, Morin et al. (51) found that 23% of COVID-19 survivors were suffering from anxiety and 7% from PTSD.

Based on our defined cut-off scores of the PCL-C and GAD-7, 11.4% of the survivors met the criteria for PTSD, and 10.3% met the criteria for anxiety, both of which are lower than those mentioned above. These lower incidences reported in our study may offer some evidence that mental disorders might be a disabling short-term consequence (i.e., within the first few months) for COVID-19 survivors and will fade over time. However, Tu et al. (52) found that the total PCL-5 scores for COVID-19 survivors increased by 20% from the 3- to 6-month follow-up, thus illustrating that PTSD symptoms may deteriorate without early intervention. In another cohort study (53) of COVID-19 intensive care unit (ICU) survivors, 23% of patients reported psychological distress at 6 weeks, with the rate remaining similar until 6 months after hospital discharge. The mental health results among COVID-19 survivors might vary slightly from study to study due to the different types of questionnaires and the medium of investigation (online or face to face), as well as the time point of follow-up, but the prevailing phenomenon of psychiatric ill health is unquestionable. A recent meta-analysis identified that during previous severe coronavirus outbreaks, the rate of survivors suffering from depression was 15% and PTSD was 33%, at a mean follow-up of 22.6 and 32.2 months, respectively (4), which might provide some evidences for the long-term perspective of mental health in terms of SARS-COV-2 infection.

Additionally, we assessed the QoL of subjects *via* the SF-36. The QoL of COVID-19 survivors has been reported in several studies, where the follow-up ranged from 1 to 6 months post-discharge (54–57). While the QoL assessment tools may vary in different studies, all have showed a decline in QoL. In a 2-month follow-up, Strumiliene et al. (56) reported a common decline among the SF-36 scores and suggested this may be due to the impaired physical functioning and emotional status, as well as the impact on lung function following SARS-COV-2 infection. Moreover, in a 6-month follow-up study (57), 52.4% of non-ICU COVID-19 survivors reported lower QoL, while the rate of ICU survivors was much higher. Compared with Chinese norms (58), we also observed a considerable reduction in QoL across all domains in both the non-PTSD and PTSD groups. The scores of physical role, mental role, and mental health were the lowest, especially the mean score of the physical role and emotional role, the mean differences of which were as high as 40 between the two groups. Our results illustrate that the QoL of COVID-19 survivors was significantly decreased even at 6 months post-discharge, and those suffering from PTSD had a much worse QoL. The relationship between PTSD and QoL is bidirectional (14). On the one hand, those with a poor QoL are more vulnerable to develop PTSD when facing traumatic events. Conversely, PTSD symptoms (i.e., re-experiencing and becoming emotionally upset due to trauma experiences) caused by SARS-COV-2 infection could trigger or worsen pre-existing physical disorders or mental health, which would lead to a further decline in QoL.

To clarify possible confounders between PTSD and factors significantly correlated with it in univariate analysis, as well as those that did not show a significant relation but play an important role in its incidence (i.e., sex, age, and comorbidities), logistic regression analysis was undertaken in our study. The results showed that PTSD at follow-up was associated with persistent respiratory symptoms, sleep difficulty, and diagnoses of anxiety. Respiratory manifestations are the primary symptoms of COVID-19 patients. Many survivors underwent respiratory suffering even several months post-discharge (59, 60). The correlation between PTSD and respiratory symptoms, as well as sleep difficulty, is bidirectional. Ongoing physical symptoms could lead to psychiatric ill health, and conversely, increased mental distress may present as physical manifestations. Moreover, sleep difficulty is not only a physical problem but also the consequence of many mental diseases (61, 62), while long-term poor sleep also leads to mental disorders (63, 64). It is widely accepted that mental distress such as anxiety and depression is highly related to PTSD (65). Our study found that people with anxiety are 15 times more likely to have PTSD than those without anxiety. This result illustrates a correlation between anxiety and PTSD, although the cause-and-effect conclusions require further exploration.

Our study features a number of limitations. Firstly, the lack of analysis on psychiatric interventions within the sample post-discharge may have led to the undervaluation of the psychological impairment in COVID-19 survivors. Furthermore, most of the recruited survivors were middle-aged and elderly, and therefore, the findings may not be relevant for younger populations. Finally, the lack of an uninfected control group from the same area may lower the persuasiveness of the study findings to some extent. Nevertheless, the advantage is that our study sample is large enough to ensure the credibility of our data.

## Conclusions

Our study has illustrated that a significant number of COVID-19 survivors are suffering from physical or mental distress to varying degrees at 6 months post-discharge. People with PTSD suffer to a greater degree from persistent respiratory symptoms and sleep difficulty, as well as anxiety and poor QoL. All these factors indicate that we should pay greater attention to the mental health of COVID-19 survivors, especially the PTSD symptoms in the early phase, which may play an important role in the recovery of both the physical and psychological heath of COVID-19 survivors. The long-term effects of SARS-COV-2 infection remain unclear at present, and therefore, longer follow-ups with larger samples are necessary.

## Data Availability Statement

The raw data supporting the conclusions of this article will be made available by the authors, without undue reservation.

## Ethics Statement

The Ethical Committee of Guangdong Provincial Hospital of Chinese Medicine reviewed and approved the study protocol (BF2020-205-01). The patients/participants provided their written informed consent to participate in this study.

## Author Contributions

ML, JH, XZ, QL, LH, XiaohX, QW, BF, and DZ contributed to the clinical data. ZH, HW, and XiaohX conducted the data management and statistical analysis. LH and XiaohX drafted the manuscript. DZ, YL, and LZ contributed to the revision of the manuscript. XiaotX and QD were responsible for supervision or mentorship. YL, QD, BD, JG, and ZZ were responsible for the research idea and interpretation of the results. All authors contributed to the article and approved the submitted version.

## Funding

This study was funded by the National Key R&D Plan of China (2020YFC0841600), the Guangdong Provincial Key Laboratory of Research on Emergency in TCM (2017B030314176), the R&D Plan in Key Areas of Guangdong Province (2020B1111300005), and the National Administration of Traditional Chinese Medicine (2020ZYLCYJ05-11).

## Conflict of Interest

The authors declare that the research was conducted in the absence of any commercial or financial relationships that could be construed as a potential conflict of interest.

## Publisher's Note

All claims expressed in this article are solely those of the authors and do not necessarily represent those of their affiliated organizations, or those of the publisher, the editors and the reviewers. Any product that may be evaluated in this article, or claim that may be made by its manufacturer, is not guaranteed or endorsed by the publisher.
