# Supplementary material for: Post-traumatic Stress Disorder Symptoms and Quality of Life of COVID-19 Survivors at 6-Month Follow-Up: A Cross-Sectional Observational Study
Source: Front Psychiatry. 2022 Jan 10;12:782478. doi: 10.3389/fpsyt.2021.782478 (PMC8784850; doi:10.3389/fpsyt.2021.782478)
Supplement: Supplementary file 1 [file Data_Sheet_1.docx]

Supplementary Material

**COVID-19 Post-discharged survey**

**Part 1 symptom questionnaire**

1. How about your current health status?

□ Same as prior to COVID-19

□ Often feel fatigue, and easier to get tired after activity now than prior to COVID-19

□ Better health condition than prior to COVID-19

2. Do you have any obvious discomfort after your COVID-19 related discharge?

□ No.

□ Yes. If yes, please answer the follow questions in detail.

3. What is your most uncomfortable feeling now compared to not infected with SARS-COV-2.

please specify

4. Have you experienced any newly onset symptoms after COVID-19 related discharge?

If yes, please specify

5. Do you have any of the following symptoms that are newly onset post COVID-19 and persistent?

□ No □ Fatigue □ Myalgia □ Joint pain □ Chills □ Sweating

□ low grade fever (37.3-38℃)

□ Any other if yes, please specify

6. How do you feel about your muscle strength compared with the status prior to COVID-19?

□ Same as before □ Worse than before □ Better than before

7. Do you have any of the following symptoms that are newly onset post COVID-19 and persistent?

□ No □ shortness of breath □ Chest distress □ Chest pain □ Cough □ Expectoration

□ Any other if yes, please specify

8. Are you more prone to suffer from the following symptoms after discharge?

□ No □ Diarrhea □ Nausea □Vomiting □Abdominal distention or bloating

9. How do you feel about your appetite compared with the status prior to COVID-19?

□ Same as before □ Worse than before □ Better than before

10. How do you feel about your vision compared with the status prior to COVID-19?

□ Same as before □ Weaker than before □ Better than before

11. How do you feel about your attention and memory compared with the status prior to COVID-19?

□ Same as before □ Worse than before □ Better than before

12. What do you think about your sleeping compared with the status prior to COVID-19?

□ Same as before □ Worse than before □ Better than before

13. Have you experienced hair loss now compared with the status prior to COVID-19?

□ No hair loss before or after COVID-19

□ Hair loss is same as before

□ Lose more hair than before

□ Lose less hair than before

**Generalized Anxiety Disorder 7-item (GAD-7) scale**

| Over the last 2 weeks, how often have you been | Not at | | Several | Over half | Nearly |
| --- | --- | --- | --- | --- | --- |
| bothered by the following problems? | all sure | | days | the days | every day |
| 1. Feeling nervous, anxious, or on edge | | 0 | 1 | 2 | 3 |
| 2. Not being able to stop or control worrying | | 0 | 1 | 2 | 3 |
| 3. Worrying too much about different things | | 0 | 1 | 2 | 3 |
| 4. Trouble relaxing | | 0 | 1 | 2 | 3 |
| 5. Being so restless that it's hard to sit still | | 0 | 1 | 2 | 3 |
| 6. Becoming easily annoyed or irritable | | 0 | 1 | 2 | 3 |
| 7. Feeling afraid as if something awful might happen | | 0 | 1 | 2 | 3 |

If you checked off any problems, how difficult have these made it for you to do your work, take care of things at home, or get along with other people?

**PTSD CheckList – Civilian Version (PCL-C)**

Below is a list of problems and complaints that people sometimes have in response to stressful life experiences. Please read each one carefully, pick the answer that indicates how much you have been bothered by that problem in the last month.

| **No.** | **Response** | **Not at all** | **A little bit** | **Moderately** | **Quite a bit** | **Extremely** |
| --- | --- | --- | --- | --- | --- | --- |
| **1.** | Repeated, disturbing memories, thoughts, or images of a stressful experience from the past? | O | O | O | O | O |
| **2.** | Repeated, disturbing dreams of a stressful experience from the past? | O | O | O | O | O |
| **3.** | Suddenly acting or feeling as if a stressful experience were happening again (as if you were reliving it)? | O | O | O | O | O |
| **4.** | Feeling very upset when something reminded you of a stressful experience from the past? | O | O | O | O | O |
| **5.** | Having physical reactions (e.g., heart pounding, trouble breathing, or sweating) when something reminded you of a stressful experience from the past? | O | O | O | O | O |
| **6.** | Avoid thinking about or talking about a stressful experience from the past or avoid having feelings related to it? | O | O | O | O | O |
| **7.** | Avoid activities or situations because they remind you of a stressful experience from the past? | O | O | O | O | O |
| **8.** | Trouble remembering important parts of a stressful experience from the past? | O | O | O | O | O |
| **9.** | Loss of interest in things that you used to enjoy? | O | O | O | O | O |
| **10.** | Feeling distant or cut off from other people? | O | O | O | O | O |
| **11.** | Feeling emotionally numb or being unable to have loving feelings for those close to you? | O | O | O | O | O |
| **12.** | Feeling as if your future will somehow be cut short? | O | O | O | O | O |
| **13.** | Trouble falling or staying asleep? | O | O | O | O | O |
| **14.** | Feeling irritable or having angry outbursts? | O | O | O | O | O |
| **15.** | Having difficulty concentrating? | O | O | O | O | O |
| **16.** | Being “super alert” or watchful on guard? | O | O | O | O | O |
| **17.** | Feeling jumpy or easily startled? | O | O | O | O | O |

**36-Item Short Form Survey Instrument（SF-36）**

Choose one option the each questionnaire item

1. In general, would you say your health is:

O excellent O Very good O Good O Fair  O Poor

2. **Compared to one year ago**, how would you rate your health in general **now**?

O Much better now than one year ago

O Somewhat better now than one year ago

O About the same

O Somewhat worse now than one year ago

O Much worse now than one year ago

The following items are about activities you might do during a typical day. Does your health now limit you in these activities? If so, how much?

|  | Yes, limited a lot | Yes, limited a little | No, not limited at all |
| --- | --- | --- | --- |
| 3. Vigorous activities, such as running, lifting heavy objects, participating in strenuous sports | O | O | O |
| **4.Moderate activities**, such as moving a table, pushing a 1 vacuum cleaner, bowling, or playing golf | O | O | O |
| 5. Lifting or carrying groceries | O | O | O |
| 6. Climbing **several** flights of stairs | O | O | O |
| 7. Climbing **one** flight of stairs | O | O | O |
| 8. Bending, kneeling, or stooping | O | O | O |
| 9. Walking **more than a mile** | O | O | O |
| 10. Walking **several blocks** | O | O | O |
| 11. Walking **one block** | O | O | O |
| 12. Bathing or dressing yourself | O | O | O |

During the past 4 weeks, have you had any of the following problems with your work or other regular daily activities as a result of your physical health?

13. Cut down the amount of time you spent on work or other activities

O Yes O No

14. Accomplished less than you would like

O Yes O No

15. Were limited in the kind of work or other activities

O Yes O No

16. Had difficulty performing the work or other activities (for example, it took extra effort)

O Yes O No

During the past 4 weeks, have you had any of the following problems with your work or other regular daily activities as a result of any emotional problems (such as feeling depressed or anxious)?

17.Cut down the **amount of time** you spent on work or other activities

O Yes O No

**18.Accomplished less** than you would like

O Yes O No

19. Didn't do work or other activities as **carefully** as usual

O Yes O No

20.During the **past 4 weeks**, to what extent has your physical health or emotional problems interfered with your normal social activities with family, friends, neighbors, or groups?

O Not at all O Moderately O Quite a bit O Extremely

21. How much **bodily** pain have you had during the **past 4 weeks**?

O None O Very mild O Mild O Moderate O Severe O Very severe

22.During the **past 4 weeks**, how much did **pain** interfere with your normal work (including both work outside the home and housework)?

O Not at all O A little bit O Moderately O Quite a bit O Extremely

These questions are about how you feel and how things have been with you **during the past 4 weeks**. For each question, please give the one answer that comes closest to the way you have been feeling.

How much of the time during the past 4weeks

|  | All of the time | Most  of the time | A good  bit of the time | Some  of the time | A little of the time | None  of the time |
| --- | --- | --- | --- | --- | --- | --- |
| 23. Did you feel full of pep? | O 1 | O 2 | O 3 | O 4 | O 5 | O 6 |
| 24. Have you been a very nervous person? | O 1 | O 2 | O 3 | O 4 | O 5 | O 6 |
| 25. Have you felt so down in the dumps that nothing could cheer you up? | O 1 | O 2 | O 3 | O 4 | O 5 | O 6 |
| 26. Have you felt calm and peaceful? | O 1 | O 2 | O 3 | O 4 | O 5 | O 6 |
| 27. Did you have a lot of energy? | O 1 | O 2 | O 3 | O 4 | O 5 | O 6 |
| 28. Have you felt downhearted and blue? | O 1 | O 2 | O 3 | O 4 | O 5 | O 6 |
| 29. Did you feel worn out? | O 1 | O 2 | O 3 | O 4 | O 5 | O 6 |
| 30. Have you been a happy person? | O 1 | O 2 | O 3 | O 4 | O 5 | O 6 |
| 31. Did you feel tired? | O 1 | O 2 | O 3 | O 4 | O 5 | O 6 |

32. During the **past 4 weeks**, how much of the time has **your physical health or emotional problems** interfered with your social activities (like visiting with friends, relatives, etc.)?

How TRUE or FALSE is **each** of the following statements for you.

|  | Definitely true | Mostly true | Don't know | Mostly false | Definitely false |
| --- | --- | --- | --- | --- | --- |
| 33. I seem to get sick a little easier than other people | O 1 | O 2 | O 3 | O 4 | O 5 |
| 34. I am as healthy as anybody I know | O 1 | O 2 | O 3 | O 4 | O 5 |
| 35. I expect my health to get worse | O 1 | O 2 | O 3 | O 4 | O 5 |
| 36. My health is excellent | O 1 | O 2 | O 3 | O 4 | O 5 |
